# Proteolysis Assays With Conserved or Aminofluorescein-Labeled Red Blood Cells

**DOI:** 10.1155/2024/7919329

**Published:** 2024-09-27

**Authors:** Mohamed K. Al-Essa, Tamara Al-Qudah, Akram Kamal A. Al Hadidi, Nida'a H. Alshubbak

**Affiliations:** ^1^ Department of Physiology and Biochemistry Faculty of Medicine The University of Jordan, Amman, Jordan; ^2^ Faculty of Medicine The University of Jordan, Amman, Jordan; ^3^ Faculty of Dentistry The University of Jordan, Amman, Jordan

**Keywords:** CBQCA, DMSO conservation, enzyme, proteolysis assay, RBC labeling, spectrofluorimetry

## Abstract

**Backgrounds:** Various physiological functions and reaction cascades, as well as disease progression in the living systems, are controlled by the activity of specific proteolytic enzymes. We conducted the study to evaluate protease activity by assessing peptide fragments from either conserved or labeled red blood cells (RBCs) with aminofluorescein (AF) in the reaction media.

**Methods:** RBCs were incubated in media containing trypsin. Subsequently, the concentration of peptide fragments in the reaction media, resulted by the digestion with trypsin from conserved cells, was estimated by 3-(4-carboxybenzoyl)quinoline-2-carboxaldehyde (CBQCA) as an amine-reactive fluorogenic reagent. In a second approach, we conjugated AF to the conserved RBCs and then exposed AF-labeled RBCs to trypsin. This was followed by directly measuring the fluorescence intensity (FI) in the reaction media to estimate the concentration of AF-labeled peptide fragments resulting from the enzyme's activity.

**Results:** Show a concentration- and time-dependent increase in FIs, reflecting the activity of trypsin as a proteolytic enzyme. The FIs increased significantly by 4 to 5 folds in samples treated with different enzyme concentrations, and by over 11 folds after 2 h incubation in media containing a 50 *μ*L trypsin, as evidenced by CBQCA assays.

**Conclusion:** These fast and affordable approaches could be applied with high reliability for the general estimation of protease activity in samples and customized for diagnostic purposes and prognostic evaluation in various diseases.

## 1. Introduction

Proteolytic enzymes play crucial roles through their catalytic activity in carrying out diverse functions in living systems and protein turnover [1]. Their activity is highly controlled, and by targeting specific substrates, proteolytic enzymes are ensuring the control of the pharmacological activity of functional peptides in various physiological and regulatory mechanisms, thereby mediating diverse vital functions in humans [2, 3]. More than 550 classified proteases have been reported in humans, performing various functions and roles in regulatory mechanisms [4]. Proteases are also involved in pathogenic conditions such as inflammatory reactions and immune responses by targeting specific bioactive protein structures to switch on reaction cascades, ensure cellular growth and tissue arrangement, and accordingly provide maintenance of structure and function [5–7]. The activity of proteases is encountered by endogenous inhibitors, which control their catalytic behavior and prevent unfavorable progression in regulatory mechanisms [8]. Uncontrolled or imbalanced activity between proteases and their inhibitors may lead to disease development and progression [9]. The increased activity of proteolytic enzymes in nephritis and inflammatory bowel diseases suggests their role in disturbing structural integrity and the cellular microenvironment, thereby, mediating tissue injury and functional dysregulation or autoimmune reactivity [10–16]. The implication of proteases in chronic inflammation was elucidated by their influence on cytokine activity, leading to tissue degeneration or uncontrolled proinflammatory responses and hindering tissue healing during the remodeling process and tissue repair [17, 18]. In the context of cancer, various proteolytic enzymes have been characterized as playing roles in tumorigenesis, angiogenesis, and the invasiveness of cancer cells [19–21]. Reports have described this phenomenon by targeting the extracellular matrix to facilitate metastasis and enhance disease progression [22–25]. The control of protease activity and targeting by inhibitors is suggested as a potential strategy in cancer therapy [26].

These studies highlight the importance of research on proteolytic enzymes to provide a comprehensive understanding of their intricate networks and the crosstalk between enzymes and their respective substrates, clarifying their potential contribution to controlling specific physiological functions as well as their implication in disease progression. Consequently, the development of methods for assessing protease activity in samples could have beneficial applications in diagnostic techniques and the evaluation of disease prognosis, as well as in the development of promising therapeutic prospects related to their dysregulation in pathological conditions.

Various analytical approaches were employed for the identification, characterization, and evaluation of proteolytic enzymes in the collected samples. Methods were based on the estimation of their hydrolytic products using spectrophotometry or spectrofluorimetry techniques by assessing neo-N-termini as indicators for proteolysis [27, 28]. For these purposes, various substrates with chromogenic or fluorogenic properties were produced by life science companies to evaluate the activity of proteolytic enzymes and indicate the catalytic outcome of proteases. In a previous study, we used fluorogenic amine-reactive dyes to estimate the increase in peptide concentration caused by the hydrolytic effect of trypsin on bovine serum albumin (BSA) [28]. Recently, fluorescence detection methods have been combined with new technology to increase the sensitivity of testing [29–31]. Isotope-labeled substrates have also been reported as useful approaches for estimating neo-N-termini and protease cleavage products [32, 33]. Other sophisticated analytical approaches by spectroscopic analysis have proven valuable for the characterization of proteolytic enzymes, profiling of substrates, and discovery of protein biomarkers [34–39]. Nowadays, researchers are seeking to combine proteomic and genomic analytical approaches and apply innovative technologies to identify specific disease biomarkers for early detection, precise diagnostic application, and prognostic or therapeutic evaluation in specific diseases [40, 41].

Human red blood cells (RBCs) possess more than 50 different types of transmembrane and peripheral proteins that contribute to their structural and functional properties [42]. Methods intended for the conservation and cryopreservation of RBCs were applied to allow longer storage times and reduce post-transfusion risks of blood cell therapy [43]. Dimethyl sulfoxide (DMSO) was reported as a cryopreservant for cells with a potential effect on the lipid bilayer membrane integrity and causing denaturation and destabilization of secondary and tertiary protein structures when used in a high concentration [44–49]. Numerous studies have demonstrated the potential modification of RBCs for therapeutic delivery and diagnostic applications [50, 51]. In previous investigations, chemical modification methods were applied to ensure the covalent binding of amine-containing products to -COOH groups on RBCs by EDC-NHS activation [52, 53]. These studies generally explore various approaches for conserving and modifying RBCs in order to investigate their potential applications in clinical therapy and diagnostic techniques.

The proteins from either conserved or labeled RBCs can be targeted and serve as substrates for the activity of proteolytic enzymes. By estimating the peptide fragments resulting from the exposure of RBCs to a protease, the activity of the enzyme can be strongly indicated. This cost-effective approach can be valuable for researchers in measuring nonspecific proteolysis in media and can be customized to estimate the activity of a particular enzyme, aiding in the comprehension of its physiological function or potential involvement in a specific disease.

## 2. Methods and Materials

### 2.1. Materials

Trypsin/EDTA 0.05%/0.02%, in PBS w/o: Ca^2+^ and Mg^2+^ (PAN Biotech, Aidenbach, Germany), 3-(4-carboxybenzoyl)quinoline-2-carboxaldehyde (CBQCA) (Invitrogen, Eugene, United States), 6-aminofluorescein (AF) (Santa Cruz Biotechnology Inc., Dallas, United States), potassium cyanide (KCN) (provided with CBQCA kit), DMSO (AppliChem, GmbH, Darmstadt, Germany), Dulbecco's phosphate-buffered saline (DPBS) w/o: Ca^2+^ and Mg^2+^ (PAN Biotech, Aidenbach, Germany), N-(3-Dimethylaminopropyl)-N′-ethylcarbodiimide hydrochloride (EDC) (Sigma-Aldrich, St. Louis, United States), N-hydroxysuccinimide (NHS) (Sigma–Aldrich, St. Louis, United States), sodium hydrogen carbonate (NaHCO3) (AppliChem, GmbH, Darmstadt, Germany), and sodium tetraborate (Na_2_B_4_O_7_.10H_2_O) (borax) (S.D. Fine-Chem Ltd, Mumbai, India).

### 2.2. Preparation of Conserved RBCs

The study was approved by the Institutional Review Board and Research Committees of The University of Jordan. Approximately 5 mL of venous blood was collected in an EDTA tube by authorized personnel from the elbow vein of a healthy volunteer. Cells were conserved in DMSO as described previously, with some modifications to the method [52, 53]. Briefly, from the collected blood, 4 mL was centrifuged in a 15 mL conical tube to remove plasma. The recovered cells were then resuspended in 8 mL of DPBS (pH = 7.4) and transferred slowly into a 50 mL conical tube containing 32 mL of DMSO for conservation, giving approximately 1:10 final dilution of RBCs from the blood of a healthy volunteer. After cooling, cells were stored at 4°C–8°C as stock of conserved cell suspension until use.

### 2.3. Labeling of RBCs With AF

**Figure 1:**
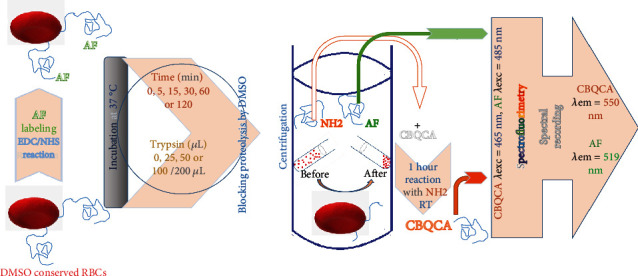
The graph displays experimental steps with unlabeled and labeled RBCs. AF labeling was performed by EDC/NHS activation to ensure covalent binding of AF to -COOH terminals. Then, either unlabeled or labeled RBCs were incubated for 1 h with 0, 25, 50, or 100 *μ*L trypsin. Another group was incubated with 50 *μ*L trypsin for 7.5, 15, 30, 60, and 120 min to study the effect of incubation time. After centrifugation, the concentration of peptide fragments resulted by the digestion with trypsin was assayed in the supernatant by CBQCA reagent for samples from unlabeled RBCs or directly for samples from AF-labeled RBCs. Fluorescence intensities were recorded by Shimadzu RF-5301PC spectrofluorometer set at *λ*exc/em = 465/550 nm for CBQCA samples and at 485/519 nm for samples from AF-labeled RBCs.

The cells were labeled with AF following activation with EDC and NHS as previously described with some modifications (Figure 1) [52, 53]. In summary, 5 mL of stock cells was centrifuged to eliminate conservation media. After resuspension in PBS, a 500 *μ*L EDC (1 M, prepared by dissolving 191 mg in 1 mL DMSO and vigorously shacked) and then 500 *μ*L NHS (2 M, prepared by dissolving 230 mg in 1 mL DMSO) were added to the 5 mL cell suspension and vortexed. After 10 min, 125 *μ*L of AF (10 mM, prepared by dissolving 3.5 mg in 1 mL DMSO) was added and vortexed, and then the volume was accomplished up to 15 mL with sodium hydrogen carbonate buffer (NaHCO3, 200 mM, pH ≈8.5, which was prepared by dissolving 0.84 g in 50 mL distilled water (DW)) to ensure better reactivity between AF with the activated –COOH groups of RBCs in an alkaline medium. The tube was protected from light and shaken at 45 RPM. After 4 h, the volume was completed to 40 mL with PBS and centrifuged to remove reaction media and excess label, followed by four washes with 40 mL PBS. Then, cells were suspended in 5 mL PBS and stored (at 4°C – 8°C) until use as stock of AF-labeled cells' suspension.

### 2.4. Incubation of Cells With Trypsin

Immediately prior to commencing the experiment, the required volume of stock cell suspensions (either unlabeled or AF-labeled) was placed in an Eppendorf tube. After removing the supernatant through centrifugation at 4500 RPM using a microcentrifuge, the cells were washed once to eliminate any potential nonspecific dissociation of the AF-dye from labeled cells in the media or to remove the DMSO media from experimental samples with conserved cells as well as any hydrolyzed protein. Subsequently, the cells were resuspended in the same volume of PBS and utilized in the experiments as follows.

#### 2.4.1. Studying the Effect of Trypsin Concentration

Two sets of four tubes each were prepared. In each tube, 25 *μ*L of cells (labeled or unlabeled) were placed in the prepared Eppendorf tubes along with 175 *μ*L of DPBS containing trypsin (0, 25, 50, or 100 *μ*L). In this context, the relative final concentrations of trypsin are 0.00625%, 0.0125%, and 0.025%, which correspond to the 12.5, 25.0, and 50.0 *μ*g of the enzyme in a 200 *μ*L reaction volume, respectively.

One set of tubes was collected at the beginning of the experiment, while the second set was collected after 1 h of incubation at 37°C. To halt the enzyme activity and collect the supernatant, 200 *μ*L of DMSO (50% in DW) was added, followed by the addition of 600 *μ*L of PBS. After cooling the cells for approximately 5–10 min in the refrigerator, a volume of 600 *μ*L of the supernatant was recovered after centrifugation at 8500 RPM.

#### 2.4.2. Studying the Effect of Incubation Time

To assess the impact of time on the hydrolytic reaction by trypsin, two quadruplet sets of unlabeled or AF-labeled RBCs were prepared. Each set consisted of five tubes. In the first set, 50 *μ*L of trypsin was prepared in 175 *μ*L PBS as above, while the second set served as controls without trypsin. After incubating with 25 *μ*L of prepared RBCs, the supernatant was collected as the above at 7.5, 15, 30, 60, and 120 min time points.

The collected supernatant samples from procedures with unlabeled cells were stored at −20°C until the CBQCA assay, while fluorescence intensities (FIs) in the supernatant of AF-labeled cells were recorded at the end of the experiment.

### 2.5. CBQCA Assays

The amine-reactive reagent 3-(4-carboxybenzoyl)quinoline-2-carboxaldehyde from the CBQCA kit was prepared according to the manufacturer's instructions. It was dissolved in DMSO at a concentration of 40 mM and stored as stock at −20°C in small vials. Prior to use, the reagent was diluted to a concentration of 2 mM in borax buffer (0.1 M, pH≈9.3, freshly prepared by dissolving 1.9 g in 50 mL DW and vigorously shacked); based on the required volume for the experiment. The KCN from the kit, which was used in the experiment, was dissolved in DW at a concentration of 20 mM and stored in the refrigerator.

To estimate the concentration of peptide fragments resulting from the hydrolytic effect of trypsin on RBCs' proteins, the collected supernatant from unlabeled cells was assayed as previously described with some modifications in the method [28]. Briefly, the protein content in 75 *μ*L of the supernatant was estimated in duplicate tubes for each collected sample. It was then adjusted to 135 *μ*L using borax buffer (0.1 M, pH≈9.3), followed by the addition of 5 *μ*L of potassium cyanide (KCN, 20 mM) and vortexing. Next, 10 *μ*L of the amine-reactive reagent (prepared from aliquoted stock in borax buffer at a concentration of 2 mM) was added and vortexed again. After 1 h of incubation in the dark with shaking at 45 RPM, the volume was adjusted to 750 *μ*L using a borax buffer.

In addition, quadruplets of trypsin at the concentrations used in our study (without cells) were prepared and assayed with CBQCA (after fixing and diluting as described above) to estimate the fluorescence signal generated by the reaction between the CBQCA reagent and trypsin (as a protein) in the media. This was performed to reflect the background signal resulting from the addition of the enzyme and to estimate the potential hydrolytic effect induced by the trypsin on proteins from RBCs.

### 2.6. Spectrofluorimetry Recording

The Shimadzu RF-5301PC spectrofluorometer (Kyōto, Japan) equipped with spectroscopy software RFPC-version 2.04 was used to measure FIs. Quantitative measurements of FIs were taken using about 400 *μ*L samples in rectangular quartz cuvettes (2 × 10 mm) with a metallic adaptor after setting the slit widths for excitation and emission at 5 nm. FIs were measured at *λ*exc/em = 465/550 nm for the CBQCA-assayed samples and 485/519 nm for the supernatant from AF-cells, based on the spectral properties of the fluorophore (Figure 1).

### 2.7. Data Collection and Analysis

Records of FIs for samples were exported to an Excel 2010 worksheet for calculation of means, standard error of the mean (SEM), and *p* values. Analyses of variance for FIs were performed by *t*-test paired sample. Differences were considered significant when the *p* values by two tails analysis were < 0.05.

## 3. Results

### 3.1. Estimation of Trypsin Activity on Unlabeled RBCs

The concentration of protein fragments hydrolyzed from incubated RBCs with varying amounts of trypsin for different durations was measured using the CBQCA assay method. The trypsin amounts used were 0, 25, 50, and 100 *μ*L for 1 h, and 50 *μ*L for 7.5, 15, 30, 60, and 120 min incubation to assess the effect of enzyme concentration, and incubation time, respectively.

#### 3.1.1. Effect of Trypsin Concentration on Unlabeled RBCs

**Figure 2:**
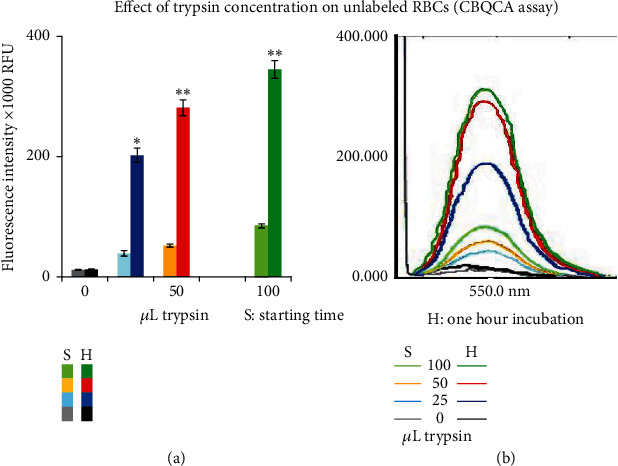
The graph displays the effect of varying trypsin concentrations on conserved RBCs. (a) Means of relative FIs (X1000) RFU obtained from the CBQCA reagent assay to reflect the concentration of peptide fragments in the collected supernatant. Significance is denoted by ^∗^ when compared to their respective controls and ^∗∗^ when significance is versus controls and samples incubated for 1 h with the 25 *μ*L trypsin after correction for the background signal generated by the reaction of added trypsin with the reagent. (b) Representative image of spectral recordings after the CBQCA assay in the supernatant from one experiment at the start of incubation (collected samples immediately after mixing) and after 1 h incubation with trypsin.

The results obtained from our study demonstrate a strong correlation and significant differences in FIs based on the concentrations of trypsin used. Initially, the mean FIs ± SEM in samples containing 25, 50, and 100 *μ*L were recorded as (39 ± 4.3, 52.3 ± 2.2, 87.2 ± 3.2)X 1000 RFU. However, after incubating the samples with the same trypsin concentrations for 1 h, the FIs increased to (202.4 ± 11.7, 281.5 ± 12.9, 344.9 ± 14.7)X 1000 RFU, respectively (*p* < 0.000002, *n* = 5, Figure 2). It is important to note that samples incubated for 1 h without trypsin did not show any change in FIs, with values of (12.8 ± 0.5 vs.11.6 ± 0.8)X 1000 RFU. However, it should be acknowledged that a portion of the fluorescence signal is attributed to the presence of trypsin in the samples. To account for this, the estimated mean FIs in samples containing 25, 50, and 100 *μ*L trypsin without RBCs were determined as (12.3 ± 0.4, 34.1 ± 0.9, and 71.5 ± 0.5)X 1000 RFU, respectively, representing the background signal induced by the addition of trypsin to the media. Therefore, by correcting the results for the background signals, the mean FI ± SEM in samples incubated for 1 h with 25, 50, and 100 *μ*L trypsin were calculated as (190.1 ± 11.7, 247.4 ± 12.9, and 273.5 ± 14.7)X 1000 RFU, respectively. These values reflect the relative increase in peptide concentrations resulting from the hydrolysis of RBCs' proteins after 1 h of incubation with the aforementioned concentrations of trypsin, respectively.

#### 3.1.2. Effect of Incubation Time of Trypsin With Unlabeled RBCs

**Figure 3:**
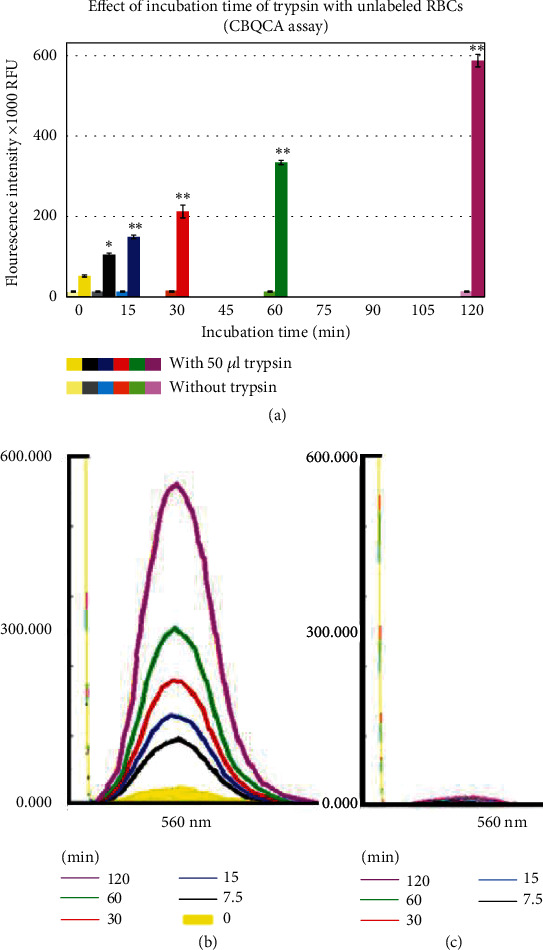
The graph displays the effect of incubation time on conserved RBCs with 50 *μ*L trypsin. (a) Means of relative FIs (X1000) RFU obtained from the CBQCA reagent assay to reflect the concentration of peptide fragments in the collected supernatant after 7.5, 15, 30, 60, and 120 min incubation. Significance is denoted by ^∗^ when compared to their respective controls and ^∗∗^ when significance is versus control and lower incubation time(s). (b, c) Representative images of spectral recordings after the CBQCA assay in the collected supernatant (with and without trypsin, respectively) at each time point.

To ensure the consistency of the results, the quadruplet sets prepared with the 50 *μ*L trypsin were used to assess the effect of the enzyme on protein degradation from the RBCs after different incubation periods of 7.5, 15, 30, 60, and 120 min. The duplicate assays for each collected supernatant from the prepared sets using CBQCA reagent revealed a time-dependent increase of FIs in samples. The FI values (± SEM) at the aforementioned time points were (105.1 ± 4.2, 149.3 ± 4.5, 212.6 ± 15.8, 334.4 ± 5.3, and 587.6 ± 15.4)X 1000 RFU, respectively (*n* = 4) (Figure 3). There were significant differences observed between any two consecutive time points (highest *p* value < 0.01, *n* = 4), as well as between the samples collected at 7.5 min and those collected at the beginning of the experiment (*p* < 0.0002, *n* = 4). Furthermore, after correcting for the background signal caused by the addition of 50 *μ*L of the enzyme, the collected samples (with trypsin) exhibited significant differences compared to their respective controls (parallel quadruplets prepared without trypsin) collected at the same time points (*p* < 0.00001, *n* = 4). The FIs in the control samples were extremely low, ranging from (13.1 ± 0.3 to 15.9 ± 0.6)X 1000 RFU, and there were no significant differences observed between any two collections at the mentioned time points.

### 3.2. Estimation of Trypsin Activity on AF-Labeled RBCs

Recording of FIs in these samples was performed directly by spectrofluorometer in the collected supernatant after incubation at the specified times as in the previous sets, without any additional procedure. Samples for estimating the effect of enzyme concentration and incubation time on AF-labeled RBCs were prepared as aforementioned with unlabeled RBCs.

#### 3.2.1. Effect of Trypsin Concentration on AF-Labeled RBCs

**Figure 4:**
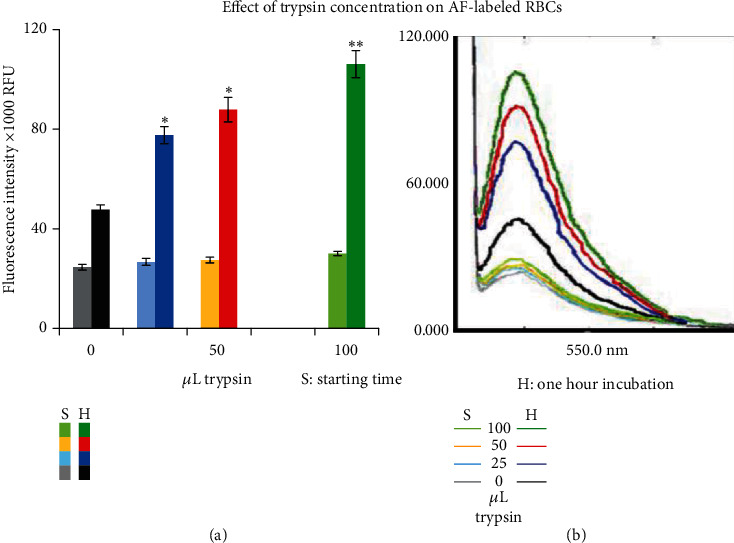
The graph displays the effect of varying trypsin concentrations on AF-Labeled RBCs. (a) Means of relative FIs (X1000) RFU by AF to reflect the concentration of labeled peptide fragments in the collected supernatant. Significance is denoted by ^∗^ when compared to their respective controls and ^∗∗^ when significance is versus controls and samples incubated for 1 h with the 25 *μ*L trypsin. (b) Representative image of spectral recordings for one experiment from the supernatant at the start of incubation (collected samples immediately after mixing) and after 1 h of incubation with trypsin.

The experiment demonstrated a relative increase in FIs after incubation with varying concentrations of trypsin. At the start of the experiment, the collected samples showed relatively low FIs (26.7 ± 1.4, 27.5 ± 1.2 and 30.1 ± 0.8)X 1000 RFU when using 25, 50 and 100 *μ*L trypsin, respectively, in the 200 *μ*L reaction volume. However, after 1 h of incubation with the aforementioned amounts of trypsin, the relative FIs in the supernatant significantly increased up to (77.7 ± 3.4, 87.9 ± 4.9 and 106.2 ± 5.4)X 1000 RFU compared to their controls (with trypsin) collected at the starting time (*p* < 0.00001, *n* = 5) as well as compared to controls (without trypsin) collected after one hour (*p* < 0.0001, *n* = 5, Figure 4). Significant differences were also found between incubated samples with 100 *μ*L versus those treated with 50 and 25 (*p* < 0.04 and < 0.003, respectively, *n* = 5). Additionally, control samples (without trypsin) also showed a significant increase in FIs after 1 h of incubation compared to those collected at the starting time (47.7 ± 2.0 vs. 24.6 ± 1.2)X 1000 RFU (*p* < 0.00001, *n* = 5). This increase in controls (without trypsin) may reflect the dissociation of the fluorophore from RBCs after 1 h of incubation at 37°C and was not promoted by an effect of trypsin on AF-labeled RBCs.

#### 3.2.2. Effect of Incubation Time of Trypsin With AF-Labeled RBCs

**Figure 5:**
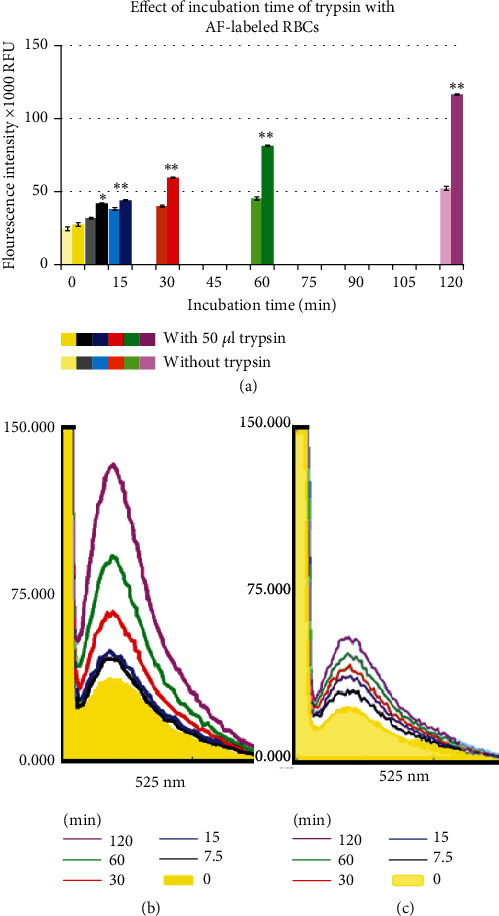
The graph displays the effect of incubation time on AF-labeled RBCs with 50 *μ*L trypsin. (a) Means of relative FIs (X1000) RFU by AF to reflect the concentration of labeled peptide fragments in the collected supernatant after 7.5, 15, 30, 60, and 120 min incubation. Significance is denoted by ^∗^ when compared to their respective controls and ^∗∗^ when significance is versus control and lower incubation time(s). (b, c) Representative images of spectral recordings in the collected supernatant (with and without trypsin, respectively) at each time point.

The effect of trypsin on AF-labeled RBCs was also assessed at various time intervals (7.5, 15, 30, 60, and 120 min) in samples incubated with 50 *μ*L of trypsin. The relative FIs in control samples (without trypsin) collected at these respective time points were (31.8 ± 0.48, 38.0 ± 0.9, 40.1 ± 0.7, 45.3 ± 1.1, and 52.2 ± 1.3)X 1000 RFU. In contrast, the recorded FIs in samples treated with trypsin showed a significant increase, reaching (41.8 ± 0.4, 44.0 ± 0.5, 59.5 ± 0.4, 81.4 ± 0.5, and 116.5 ± 0.5)X 1000 RFU compared to the control samples collected at the mentioned time points (*p* < 0.001, *n* = 4, Figure 5). Significant differences were observed between the trypsin-treated samples collected at 15 min and 7.5 min (*p* < 0.02, *n* = 4), as well as between any two other collections at the specified times (*p* < 0.000002, *n* = 4). Although minor variations in FIs were noted among the control samples (without trypsin), significant differences were also observed between samples collected at 2 h and 1 h, 1 h and 30 min and 15 and 7.5 min (*p* < 0.01, *n* = 4). These findings are further supporting our hypothesis of fluorophore dissociation from AF-labeled RBCs by a phenomenon not attributed to trypsin activity.

## 4. Discussion

Our research has focused on the potential use of DMSO-conserved RBCs to measure the activity of proteolytic enzymes in samples. The substrate for the enzyme was the proteins of RBCs. The purpose of conservation was to maintain the stability of RBCs as a stock for long-term use in research activities and, therefore, to ensure consistency of results. In stored cells and during experimental procedures (with either unlabeled or labeled), we have not observed any change in the color of the supernatant, which suggests no lyses of cells or hemoglobin degradation has taken place. Previous studies have shown that DMSO conservation can result in protein denaturation [46–49], which may benefit by suggesting more proteolytic activity of the digestive enzyme on the denatured proteins of RBCs [54]. In a previous study, the CBQCA assay was used to evaluate the activity of trypsin on BSA as a native protein [28]. Although the optimal pH for enzyme activity was respected, only minor differences were observed in that study between concentration and time groups in samples treated with trypsin. Therefore, we anticipate superior effects from trypsin on denatured RBCs' proteins when using DMSO-conserved RBCs.

Protein assay methods relying on the assessment of N-termini by chromogenic or fluorogenic reagents were suggested for the estimation of protease activity [27, 28]. In this context, spectrofluorimetry techniques have been found to be more sensitive than colorimetric methods for protein estimation, as indicated by their lower detection limits [29]. In a previous investigation, despite utilizing highly sensitive fluorogenic reagents with nearly identical detection thresholds to quantify proteins, we noted a distinction in the estimation of proteolysis when comparing amine-reactive dyes to nonamine-reactive reagent [28]. Recently, combined biosensors with amine-reactive fluorogenic reagents in chemiluminescence chips have shown the ability to precisely measure protein concentrations as low as 9 pg/mL in samples [29]. In another study, high analytical performance was achieved by employing magnetic nanoparticles, reaching a 4-pg/mL limit of detection for caspase enzyme in reaction media containing fluorescent labeled specific peptide substrate [30]. Accordingly, the utilization of fluorescence methods for detecting protease activity could have a promising potential when integrated with advanced techniques based on nanotechnology and sensors [31].

The initial approach in this study involved the utilization of CBQCA as an amine-reactive reagent to estimate the protein fragments resulting from trypsin treatment in the supernatant. The low fluorescence signal of the unconjugated reagent, along with the significant shift in spectral properties upon conjugation with the amine, makes it a highly suitable and reliable technique for determining the concentration of amine groups in the supernatant. This concentration can serve as an indicator of protein degradation from the RBCs due to the activity of trypsin. Our findings clearly demonstrate significant differences between samples containing varying concentrations of trypsin after 1 h of incubation compared to those collected at the beginning of the experiment. In contrast, control samples (without trypsin) collected after 1 h showed no difference compared to those collected at the starting time. The FI increased approximately 4–5 times with trypsin treatment, indicating the degradation of RBCs' proteins by the enzyme. Additionally, our results show that control samples (with trypsin) collected at the start of the experiment also exhibited a significant increase in FIs with increasing trypsin concentration. This increase suggests that the added enzyme may have been included as a protein in the assay, yielding an additional fluorescence signal upon reaction with CBQCA. To account for this potential error, we conducted quadruplet samples containing the applied concentrations of the enzyme in the reaction buffer without RBCs. We recorded the FIs of these samples, which reflected the background signal induced by the addition of trypsin. After correcting for the background signal, we still observed significant differences in FIs between all controls (with trypsin) collected at the start of the experiment (26.7 ± 4.3, 18.2 ± 2.2, 15.7 ± 3.2)X 1000 RFU and the corrected controls (without trypsin) for the background signal from blank samples with the added CBQCA reagent ((5.3 ± 0.8)X 1000 RFU, *p* < 0.02, *n* = 5). These differences could be attributed to the starting activity of the enzyme during the gap of time between the addition of trypsin and getting samples collected. Nevertheless, no significant difference was observed between any two groups of control samples treated with trypsin and collected at the start of the experiment. However, samples treated with 100 and 50 *μ*L trypsin and collected after 1 h showed significant increases in FI by 44% and 30%, respectively, versus those incubated with the 25 *μ*L (*p* < 0.005 and < 0.02, respectively, Figure 2). These results clearly indicate with high confidence the concentration-dependent increase of peptide fragments in the supernatant was induced by the hydrolytic activity of trypsin on RBCs' proteins upon incubation for 1 h.

The incubation time was also found to have an impact on the increase in FIs. Samples collected at various time intervals (7.5, 15, 30, 60, and 120 min) using the same concentration of trypsin (50 *μ*L) showed a significant increase in FIs (2.0, 2.9, 4.1, 6.4, and 11.2 folds (Figure 3), respectively, compared to those collected at the start of the experiment. The FI values also showed significant differences between any two closed collection times (*p* values were < 0.0002 (for the 7.5 min vs. those collected at the starting time), < 0.0005 (for the 15 vs. 7.5 min), < 0.01 (for the 30 vs. 15 min), < 0.0005 (for the 60 vs. 30 min), and < 0.00001 (for the 120 vs. 60 min), *n* = 4). These results indicate the reliability of the method and suggest an increase in the concentration of products containing amine in the collected supernatant due to the proteolytic effect of trypsin on RBCs' proteins during the reaction time.

Additionally, labeled RBCs with AF were also found to be useful for estimating trypsin activity. By labeling RBCs, it was suggested to estimate the concentration of generated peptide fragments possessing an AF label directly by estimating the fluorescein signal in the supernatant of samples upon incubation with trypsin without applying further procedures.

Results obtained from AF-labeled RBCs have demonstrated a significant increase in FI signal after 1 h of incubation with trypsin. The fold increase in FI signal was approximately 2.8, 3.3, and 3.4 when incubated with 25, 50, and 100 *μ*L trypsin, respectively, compared to their respective controls at the starting time (*p* < 0.00001, Figure 4). It is worth noting that the AF-labeled cells were thoroughly washed before each experiment to minimize any fluorescence signal in the supernatant resulting from fluorophore dissociation during storage. However, even without trypsin treatment, the control samples still exhibited a significantly higher FI signal of approximately 94% (*p* < 0.00001) after 1 h of incubation. This can be attributed to the dissociation of the fluorophore caused by the temperature effect during the incubation period, as the samples were exposed to 37°C for 1 h. To further analyze the trypsin effect, we compared the FI signal in samples treated with trypsin to their respective controls (without trypsin) after 1 h of incubation. The results showed a significantly higher FI signal of approximately 63%, 84%, and 122% in samples incubated with 25, 50, and 100 *μ*L of trypsin, respectively (*p* < 0.0001, *n* = 5) (Figure 4). Moreover, significant differences were also observed between samples treated with 100 *μ*L of trypsin compared to those treated with 50 and 25 *μ*L (*p* < 0.04 and < 0.003, respectively). This concentration-dependent increase in FI signal can be attributed to the activity of trypsin and reflects the concentration of AF-labeled fragments resulting from the hydrolytic effect of trypsin on AF-labeled RBCs.

Additionally, the incubation time of trypsin with AF-labeled RBCs also influenced the FI values. The results showed that the FI values of AF-labeled RBCs increase by approximately 31%, 16%, 49%, 80%, and 123% in samples incubated with 50 *μ*L trypsin for 7.5, 15, 30, 60, and 120 min, respectively, compared to their controls (without trypsin) collected at the mentioned time points (*p* < 0.001, *n* = 4) (Figure 5). The increase is indicative of the hydrolytic effect induced by trypsin, which accumulates over time, as evidenced by the increase in AF-labeled peptide fragments in the collected supernatant. Furthermore, there is a significant difference between any two closed collections treated with the enzyme (the highest *p* value was < 0.02 for samples collected at 15 vs. 7.5 min) (Figure 5).

Our experiment is aimed at streamlining procedures and saving time by using AF-labeled RBCs. However, we are encountering challenges in achieving higher accuracy due to the nonspecific dissociation of the fluorophore from the modified RBCs, which is not related to enzyme activity. To address this issue, we need to focus on developing a modification or labeling method that facilitates fluorophore conjugation without being affected by reaction conditions and with a lower potential for noncovalent binding to other plasma membrane structures.

Both of the techniques described here could be valuable for estimating the proteolytic activity of enzymes in samples. While using AF-labeled RBCs allows us to save time by recording FI immediately after the procedure, the CBQCA method appears to have superior sensitivity when estimating fragments in the supernatant. This is evident from the higher slope of increasing FI with doubling the concentration or reaction time compared to AF-labeled RBCs. This difference in FI increase rate could be attributed to the cleavage of proteins from conserved RBCs by trypsin at multiple sites, resulting in more amine termini being liberated. Additionally, fragmenting peptides on RBCs with initially free amine and carboxyl termini also contribute to the higher increase in FI observed with the CBQCA reagent. In contrast, when using AF-labeled RBCs, only one labeled fragment is freed to yield a relative fluorescent signal from each targeted RBC's protein with the fluorophore at its carboxyl terminus. Furthermore, a disadvantage of using AF-labeled cells is the observed nonspecific dissociation of the fluorophore, which becomes more apparent over time in control samples without trypsin. On the other hand, CBQCA control samples show slight but not significant variations in FI after correcting for the background signal caused by the addition of trypsin. This observation highlights the superiority of the CBQCA assay in estimating fragments over the use of AF-labeled RBCs in biological samples. However, researchers must be cautious when using AF-labeled RBCs to reduce testing efforts, as the nonspecific dissociation during incubation time, which is prone to misinterpretation of results.

## 5. Conclusion

Using conserved RBCs, it is possible to estimate proteolytic enzyme activity with a high level of confidence, as demonstrated in this study. The effect of trypsin on RBCs was successfully demonstrated by analyzing the concentration of the hydrolyzed peptides from RBCs' protein in the supernatant. By utilizing labeled RBCs, enzyme activity can be rapidly analyzed. However, optimization may be required to minimize errors caused by the nonspecific dissociation of the label from RBCs. The described methods are reliable for application by the researchers as well as for potential development to evaluate the activity of proteolytic enzymes in biological samples.

## Data Availability

The data that support the findings of this study are available upon reasonable request from the corresponding author.
